# 2-Amino-5-chloro­pyridinium 2-carb­oxy­benzoate–benzene-1,2-dicarb­oxy­lic acid (3/1)

**DOI:** 10.1107/S1600536810035853

**Published:** 2010-09-11

**Authors:** Madhukar Hemamalini, Hoong-Kun Fun

**Affiliations:** a X-ray Crystallography Unit, School of Physics, Universiti Sains Malaysia, 11800 USM, Penang, Malaysia

## Abstract

The asymmetric unit of the title compound, 3C_5_H_6_ClN_2_^+^·3C_8_H_5_O_4_^−^·C_8_H_6_O_4_, contains three independent 2-amino-5-chloro­pyridinium cations, three independent hydrogen phthal­ate anions and one phthalic acid mol­ecule. In the crystal structure, there are two kinds of supra­molecular tapes. One is formed by two independent cations with two anions through N—H⋯O and C—H⋯O hydrogen bonds. Another one is formed by the other cation and anion, and the phthalic acid mol­ecule *via* N—H⋯O, O—H⋯O and C—H⋯O hydrogen bonds. These two tapes are connected by an O—H⋯O hydrogen bond, forming a double-tape structure.

## Related literature

For details of hydrogen bonding, see: Jeffery (1997 ▶). For details of structures incorporating phthalic acid, see: Dale *et al.* (2004 ▶); Ballabh *et al.* (2005 ▶). For hydrogen-bond motifs, see: Bernstein *et al.* (1995 ▶). For the stability of the temperature controller used in the data collection, see: Cosier & Glazer (1986 ▶).
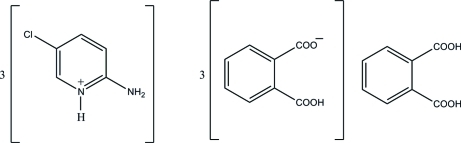


## Experimental

### 

#### Crystal data

3C_5_H_6_ClN_2_^+^·3C_8_H_5_O_4_^−^·C_8_H_6_O_4_
*M* *_r_* = 1050.19
Triclinic, 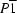

*a* = 9.8522 (2) Å
*b* = 14.0242 (2) Å
*c* = 17.4312 (3) Å
α = 68.920 (1)°
β = 87.507 (1)°
γ = 83.906 (1)°
*V* = 2234.54 (7) Å^3^
*Z* = 2
Mo *K*α radiation
μ = 0.29 mm^−1^
*T* = 100 K
0.49 × 0.22 × 0.22 mm

#### Data collection

Bruker SMART APEXII CCD area-detector diffractometer
Absorption correction: multi-scan (*SADABS*; Bruker, 2009 ▶) *T*_min_ = 0.870, *T*_max_ = 0.940
63061 measured reflections
12966 independent reflections
11359 reflections with *I* > 2σ(*I*)
*R*_int_ = 0.032

#### Refinement

*R*[*F* ^2^ > 2σ(*F* ^2^)] = 0.036
*wR*(*F* ^2^) = 0.101
*S* = 1.03
12966 reflections
696 parameters
H atoms treated by a mixture of independent and constrained refinement
Δρ_max_ = 0.44 e Å^−3^
Δρ_min_ = −0.35 e Å^−3^

### 

Data collection: *APEX2* (Bruker, 2009 ▶); cell refinement: *SAINT* (Bruker, 2009 ▶); data reduction: *SAINT*; program(s) used to solve structure: *SHELXTL* (Sheldrick, 2008 ▶); program(s) used to refine structure: *SHELXTL*; molecular graphics: *SHELXTL*; software used to prepare material for publication: *SHELXTL* and *PLATON* (Spek, 2009 ▶).

## Supplementary Material

Crystal structure: contains datablocks global, I. DOI: 10.1107/S1600536810035853/is2597sup1.cif

Structure factors: contains datablocks I. DOI: 10.1107/S1600536810035853/is2597Isup2.hkl

Additional supplementary materials:  crystallographic information; 3D view; checkCIF report

## Figures and Tables

**Table 1:** Hydrogen-bond geometry (Å, °)

*D*—H⋯*A*	*D*—H	H⋯*A*	*D*⋯*A*	*D*—H⋯*A*
O2*C*—H2*C*⋯O3*C*	0.82	1.58	2.4004 (13)	177
O2*A*—H1*OA*⋯O3*A*	0.86	1.55	2.4123 (13)	177
O2*B*—H1*OB*⋯O3*B*	0.89	1.52	2.4126 (14)	178
O2*X*—H1*OX*⋯O4*C*	0.88 (2)	1.76 (2)	2.6091 (12)	163 (2)
O4*X*—H2*OX*⋯O1*A*^i^	0.83 (2)	1.86 (2)	2.6880 (13)	177 (2)
N2*A*—H2*NA*⋯O1*B*^ii^	0.895 (18)	2.018 (18)	2.9095 (14)	173.4 (19)
N1*B*—H1*NB*⋯O4*A*^iii^	0.92 (2)	1.69 (2)	2.5938 (14)	169 (2)
N2*B*—H2*NB*⋯O3*A*^iii^	0.87 (2)	2.12 (2)	2.9570 (14)	161.8 (17)
N1*C*—H1*NC*⋯O4*B*	0.94 (2)	1.69 (2)	2.6169 (16)	168 (2)
N2*C*—H3*NC*⋯O3*B*	0.83 (2)	2.11 (2)	2.9279 (15)	168.4 (19)
C4*A*—H4*AA*⋯O2*B*^ii^	0.93	2.26	3.1614 (14)	164

Symmetry codes: (i) 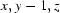; (ii) 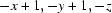; (iii) 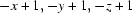.

**Fig. 1.**
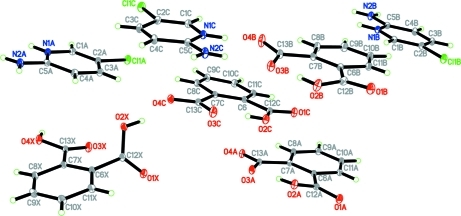
The asymmetric unit of the title compound. Displacement ellipsoids are drawn at the 30% probability level.

**Fig. 2.**
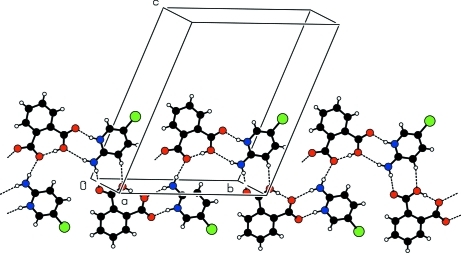
A view of supramolecular tape formed by cations (A & B) with the anions (B & C) through N—H···O and C—H···O hydrogen bonds.

**Fig. 3.**
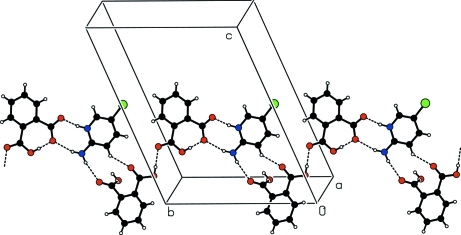
A view of supramolecular tape formed by cation B, the anion A and the phthalic acid molecule via N—H···O, O—H···O and C—H···O hydrogen bonds.

**Fig. 4.**
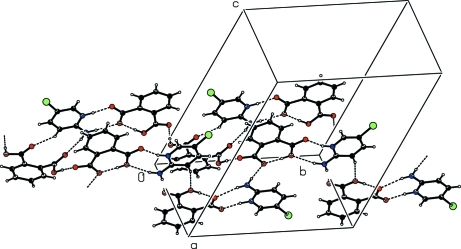
Hydrogen bonding patterns in (I).

## supplementary crystallographic information

### Comment

Hydrogen bonding is one of the key interactions for molecular assembly and molecular recognition (Jeffery, 1997).  In nature, biopolymers such as nucleic acids and polypeptides form supramolecular structures through hydrogen bonding of which the dynamic nature of the interaction contributes to chemical and biological molecular processes.  Phthalic acid forms hydrogen phthalate salts with various organic compounds. Hydrogen phthalates also form supramolecular assemblies, such as extended chains, ribbons and three-dimensional networks (Dale *et al.*, 2004; Ballabh *et al.*, 2005).  In this paper, the hydrogen-bonding patterns of tris(2-amino-5-chloropyridinium hydrogen phthalate) phthalic acid, (I), are discussed.

The asymmetric unit of the title compound consists of three crystallographically independent 2-amino-5-chloropyridinium cations (A, B & C), three hydrogen phthalate anions (A, B & C) and one phthalic acid molecule (Fig. 1). Each 2-amino-5-chloropyridinium cation is planar, with a maximum deviation of 0.008 (1) Å for atom C4A (molecule A), 0.019 (1) Å for atom Cl1B (molecule B) and 0.028 (1) Å for atom N2C (molecule C).

In the crystal structure, there are two kinds of supramolecular tapes. One is formed by the cations A and B with the anions B and C, through N—H···O and C—H···O hydrogen bonds (Fig. 2).  The another one is formed by the cation B, the anion A and the phthalic acid molecule via N—H···O, O—H···O and C—H···O hydrogen bonds (Fig. 3). Furthermore, these two tapes are connected by an O—H···O hydrogen bond, forming a double tape structure (Fig. 4). There is an intramolecular O—H···O hydrogen bond in the hydrogen phthalate anions, which generates an *S*(6) (Bernstein *et al.*, 1995) ring motif.

### Experimental

A hot methanol solution (20 ml) of 2-amino-5-chloropyridine (65 mg, Aldrich) and phthalic acid (83 mg, Merck) were mixed and warmed over a heating magnetic stirrer hotplate for a few minutes. The resulting solution was allowed to cool slowly at room temperature and yellow coloured crystals of the title compound appeared after a few days.

### Refinement

Atoms H1OX, H2OX, H1NA, H2NA, H3NA, H1NB, H2NB, H3NB, H1NC, H2NC and H3NC were located from a difference Fourier map and were refined freely [N—H = 0.83 (2)–0.92 (2) Å and O—H =0.83 (2)–0.88 (2) Å]. The remaining O-bound H atoms were also located in a difference Fourier map. They were treated as riding and the isotropic displacement parameters were refined. The C-bound H atoms were positioned geometrically [C—H = 0.93 Å] and were refined using a riding model, with *U*_iso_(H) = 1.2*U*_eq_(C).

### Crystal data

**Table:**

3C_5_H_6_ClN_2_^+^·3C_8_H_5_O_4_^−^·C_8_H_6_O_4_	*Z* = 2
*M**_r_* = 1050.19	*F*(000) = 1084
Triclinic, *P*1	*D*_x_ = 1.561 Mg m^−^^3^
Hall symbol: -P 1	Mo *K*α radiation, λ = 0.71073 Å
*a* = 9.8522 (2) Å	Cell parameters from 9964 reflections
*b* = 14.0242 (2) Å	θ = 2.4–36.1°
*c* = 17.4312 (3) Å	µ = 0.29 mm^−^^1^
α = 68.920 (1)°	*T* = 100 K
β = 87.507 (1)°	Block, yellow
γ = 83.906 (1)°	0.49 × 0.22 × 0.22 mm
*V* = 2234.54 (7) Å^3^	

### Data collection

**Table:**

Bruker SMART APEXII CCD area-detector diffractometer	12966 independent reflections
Radiation source: fine-focus sealed tube	11359 reflections with *I* > 2σ(*I*)
graphite	*R*_int_ = 0.032
φ and ω scans	θ_max_ = 30.0°, θ_min_ = 1.6°
Absorption correction: multi-scan (*SADABS*; Bruker, 2009)	*h* = −13→13
*T*_min_ = 0.870, *T*_max_ = 0.940	*k* = −19→19
63061 measured reflections	*l* = −24→23

### Refinement

**Table:**

Refinement on *F*^2^	Primary atom site location: structure-invariant direct methods
Least-squares matrix: full	Secondary atom site location: difference Fourier map
*R*[*F*^2^ > 2σ(*F*^2^)] = 0.036	Hydrogen site location: inferred from neighbouring sites
*wR*(*F*^2^) = 0.101	H atoms treated by a mixture of independent and constrained refinement
*S* = 1.03	*w* = 1/[σ^2^(*F*_o_^2^) + (0.0547*P*)^2^ + 0.8733*P*] where *P* = (*F*_o_^2^ + 2*F*_c_^2^)/3
12966 reflections	(Δ/σ)_max_ = 0.001
696 parameters	Δρ_max_ = 0.44 e Å^−^^3^
0 restraints	Δρ_min_ = −0.35 e Å^−^^3^

### Special details

**Table:**

Experimental. The crystal was placed in the cold stream of an Oxford Cryosystems Cobra open-flow nitrogen cryostat (Cosier & Glazer, 1986) operating at 100.0 (1) K.
Geometry. All s.u.'s (except the s.u. in the dihedral angle between two l.s. planes) are estimated using the full covariance matrix. The cell s.u.'s are taken into account individually in the estimation of s.u.'s in distances, angles and torsion angles; correlations between s.u.'s in cell parameters are only used when they are defined by crystal symmetry. An approximate (isotropic) treatment of cell s.u.'s is used for estimating s.u.'s involving l.s. planes.
Refinement. Refinement of F^2^ against ALL reflections. The weighted R-factor wR and goodness of fit S are based on F^2^, conventional R-factors R are based on F, with F set to zero for negative F^2^. The threshold expression of F^2^ > 2σ(F^2^) is used only for calculating R-factors(gt) etc. and is not relevant to the choice of reflections for refinement. R-factors based on F^2^ are statistically about twice as large as those based on F, and R- factors based on ALL data will be even larger.

### Fractional atomic coordinates and isotropic or equivalent isotropic displacement parameters (Å^2^)

**Table:**

	*x*	*y*	*z*	*U*_iso_*/*U*_eq_	
Cl1A	0.45378 (3)	0.01683 (2)	0.401827 (17)	0.02226 (7)	
N1A	0.42388 (10)	−0.14773 (7)	0.26255 (6)	0.01629 (18)	
N2A	0.37929 (11)	−0.11497 (8)	0.12618 (6)	0.02002 (19)	
C1A	0.44162 (11)	−0.11900 (9)	0.32770 (7)	0.0169 (2)	
H1AA	0.4604	−0.1687	0.3793	0.020*	
C2A	0.43208 (11)	−0.01796 (9)	0.31773 (7)	0.0166 (2)	
C3A	0.40361 (12)	0.05652 (9)	0.23923 (7)	0.0186 (2)	
H3AA	0.3962	0.1260	0.2317	0.022*	
C4A	0.38690 (12)	0.02632 (9)	0.17425 (7)	0.0186 (2)	
H4AA	0.3694	0.0752	0.1222	0.022*	
C5A	0.39631 (11)	−0.07983 (9)	0.18631 (7)	0.0160 (2)	
O1A	0.10557 (9)	0.89607 (6)	0.30990 (5)	0.02235 (18)	
O2A	0.12677 (9)	0.75913 (7)	0.27580 (5)	0.02175 (17)	
H1OA	0.1241	0.6937	0.2990	0.074 (8)*	
O3A	0.11516 (9)	0.57696 (6)	0.34407 (5)	0.02090 (17)	
O4A	0.13550 (9)	0.45909 (6)	0.46883 (5)	0.01945 (16)	
C6A	0.15173 (11)	0.73752 (8)	0.42026 (7)	0.01448 (19)	
C7A	0.15713 (11)	0.62917 (8)	0.45923 (7)	0.01426 (19)	
C8A	0.18655 (12)	0.58619 (9)	0.54319 (7)	0.0173 (2)	
H8AA	0.1900	0.5153	0.5691	0.021*	
C9A	0.21081 (12)	0.64477 (9)	0.58947 (7)	0.0188 (2)	
H9AA	0.2309	0.6136	0.6451	0.023*	
C10A	0.20461 (11)	0.75089 (9)	0.55149 (7)	0.0181 (2)	
H10C	0.2203	0.7916	0.5816	0.022*	
C11A	0.17475 (11)	0.79561 (8)	0.46828 (7)	0.0166 (2)	
H11C	0.1699	0.8667	0.4435	0.020*	
C12A	0.12482 (11)	0.80204 (9)	0.33027 (7)	0.0169 (2)	
C13A	0.13380 (11)	0.55001 (8)	0.42119 (7)	0.0159 (2)	
Cl1B	0.83721 (3)	0.98373 (2)	0.460859 (17)	0.02238 (7)	
N1B	0.87824 (10)	0.68696 (7)	0.58805 (6)	0.01643 (18)	
N2B	0.92305 (11)	0.58396 (8)	0.72337 (6)	0.0204 (2)	
C1B	0.85787 (11)	0.77934 (9)	0.52648 (7)	0.0168 (2)	
H1BA	0.8419	0.7824	0.4734	0.020*	
C2B	0.86063 (11)	0.86783 (8)	0.54165 (7)	0.0167 (2)	
C3B	0.88534 (12)	0.86258 (9)	0.62235 (7)	0.0180 (2)	
H3BA	0.8876	0.9223	0.6337	0.022*	
C4B	0.90591 (12)	0.76850 (9)	0.68346 (7)	0.0180 (2)	
H4BA	0.9224	0.7641	0.7368	0.022*	
C5B	0.90231 (11)	0.67760 (9)	0.66603 (7)	0.0163 (2)	
O1B	0.69143 (10)	0.98082 (7)	0.04092 (6)	0.02612 (19)	
O2B	0.65261 (11)	0.84422 (7)	0.01512 (5)	0.02608 (19)	
H1OB	0.6605	0.7760	0.0394	0.063 (7)*	
O3B	0.66767 (10)	0.65998 (7)	0.08159 (5)	0.02202 (17)	
O4B	0.74305 (12)	0.54133 (7)	0.19770 (6)	0.0299 (2)	
C6B	0.72617 (11)	0.82179 (8)	0.15300 (7)	0.0158 (2)	
C7B	0.73424 (11)	0.71332 (8)	0.19160 (7)	0.0161 (2)	
C8B	0.76723 (13)	0.67044 (9)	0.27519 (7)	0.0208 (2)	
H8BA	0.7731	0.5994	0.3009	0.025*	
C9B	0.79150 (13)	0.72924 (10)	0.32115 (8)	0.0229 (2)	
H9BA	0.8115	0.6982	0.3768	0.027*	
C10B	0.78571 (13)	0.83507 (9)	0.28324 (8)	0.0218 (2)	
H10D	0.8029	0.8757	0.3131	0.026*	
C11B	0.75399 (12)	0.87967 (9)	0.20027 (7)	0.0189 (2)	
H11D	0.7511	0.9506	0.1751	0.023*	
C12B	0.68907 (12)	0.88739 (9)	0.06458 (7)	0.0191 (2)	
C13B	0.71365 (12)	0.63318 (9)	0.15401 (7)	0.0184 (2)	
Cl1C	0.74116 (3)	0.09871 (2)	0.262958 (19)	0.02519 (7)	
N1C	0.71246 (10)	0.39640 (8)	0.14020 (6)	0.01702 (18)	
N2C	0.66401 (12)	0.50347 (8)	0.00715 (6)	0.0212 (2)	
C1C	0.73257 (12)	0.30282 (9)	0.20019 (7)	0.0179 (2)	
H1CA	0.7586	0.2975	0.2523	0.022*	
C2C	0.71499 (12)	0.21651 (9)	0.18475 (7)	0.0179 (2)	
C3C	0.67473 (12)	0.22535 (9)	0.10550 (7)	0.0202 (2)	
H3CA	0.6610	0.1671	0.0943	0.024*	
C4C	0.65611 (12)	0.32028 (9)	0.04550 (7)	0.0198 (2)	
H4CA	0.6296	0.3269	−0.0069	0.024*	
C5C	0.67722 (11)	0.40911 (9)	0.06294 (7)	0.0167 (2)	
O1C	0.43733 (9)	0.64971 (6)	0.32698 (5)	0.02123 (17)	
O2C	0.40372 (9)	0.63362 (6)	0.20871 (5)	0.02070 (17)	
H2C	0.4007	0.5875	0.1905	0.031*	
O3C	0.38734 (10)	0.49905 (7)	0.15591 (5)	0.02425 (18)	
O4C	0.38122 (9)	0.33117 (7)	0.19965 (5)	0.02149 (17)	
C6	0.43344 (10)	0.47894 (8)	0.33267 (7)	0.01398 (19)	
C7C	0.42704 (11)	0.39947 (8)	0.30070 (7)	0.01478 (19)	
C8C	0.44379 (11)	0.29758 (9)	0.35547 (7)	0.0178 (2)	
H8CA	0.4429	0.2449	0.3349	0.021*	
C9C	0.46168 (12)	0.27235 (9)	0.43908 (7)	0.0197 (2)	
H9CA	0.4730	0.2039	0.4738	0.024*	
C10C	0.46265 (12)	0.34990 (9)	0.47055 (7)	0.0203 (2)	
H10B	0.4716	0.3341	0.5268	0.024*	
C11C	0.45017 (11)	0.45127 (9)	0.41744 (7)	0.0173 (2)	
H11B	0.4530	0.5028	0.4390	0.021*	
C12C	0.42405 (11)	0.59425 (8)	0.28679 (7)	0.01572 (19)	
C13C	0.39680 (11)	0.41033 (9)	0.21310 (7)	0.0163 (2)	
O1X	0.04664 (9)	0.38078 (6)	0.11892 (5)	0.02047 (17)	
O2X	0.23396 (8)	0.28591 (6)	0.09895 (5)	0.01790 (16)	
O3X	0.06412 (10)	0.13221 (6)	0.19699 (5)	0.02195 (17)	
O4X	0.05428 (10)	0.00430 (6)	0.15024 (6)	0.02134 (17)	
C6X	0.03190 (11)	0.27963 (8)	0.03490 (7)	0.01521 (19)	
C7X	0.01326 (11)	0.17532 (8)	0.05616 (7)	0.01504 (19)	
C8X	−0.04564 (11)	0.14237 (9)	−0.00028 (7)	0.0180 (2)	
H8XA	−0.0590	0.0735	0.0138	0.022*	
C9X	−0.08435 (12)	0.21229 (10)	−0.07743 (7)	0.0216 (2)	
H9XA	−0.1205	0.1898	−0.1156	0.026*	
C10X	−0.06893 (13)	0.31588 (10)	−0.09748 (7)	0.0222 (2)	
H10A	−0.0970	0.3627	−0.1486	0.027*	
C11X	−0.01162 (12)	0.34997 (9)	−0.04143 (7)	0.0191 (2)	
H11A	−0.0024	0.4194	−0.0549	0.023*	
C12X	0.10282 (11)	0.31957 (8)	0.09010 (6)	0.01542 (19)	
C13X	0.04723 (11)	0.10283 (8)	0.14117 (7)	0.01508 (19)	
H1OX	0.2711 (19)	0.3112 (15)	0.1314 (12)	0.041 (5)*	
H2OX	0.070 (2)	−0.0309 (16)	0.1993 (13)	0.041 (5)*	
H1NA	0.4293 (17)	−0.2157 (14)	0.2731 (11)	0.030 (4)*	
H2NA	0.3604 (17)	−0.0697 (14)	0.0756 (11)	0.032 (4)*	
H3NA	0.3800 (17)	−0.1818 (14)	0.1364 (11)	0.030 (4)*	
H1NB	0.880 (2)	0.6302 (16)	0.5737 (13)	0.048 (6)*	
H2NB	0.9243 (18)	0.5284 (15)	0.7124 (11)	0.033 (4)*	
H3NB	0.9351 (18)	0.5837 (14)	0.7723 (12)	0.030 (4)*	
H1NC	0.7207 (19)	0.4549 (15)	0.1536 (13)	0.044 (5)*	
H2NC	0.6456 (18)	0.5087 (14)	−0.0434 (12)	0.034 (5)*	
H3NC	0.6673 (18)	0.5536 (15)	0.0214 (12)	0.034 (5)*	

### Atomic displacement parameters (Å^2^)

**Table:**

	*U*^11^	*U*^22^	*U*^33^	*U*^12^	*U*^13^	*U*^23^
Cl1A	0.03296 (15)	0.01957 (13)	0.01586 (12)	−0.00135 (11)	−0.00293 (10)	−0.00829 (10)
N1A	0.0208 (4)	0.0126 (4)	0.0151 (4)	−0.0025 (3)	−0.0013 (3)	−0.0041 (3)
N2A	0.0270 (5)	0.0179 (5)	0.0163 (5)	−0.0040 (4)	−0.0025 (4)	−0.0067 (4)
C1A	0.0200 (5)	0.0158 (5)	0.0134 (5)	−0.0023 (4)	−0.0008 (4)	−0.0033 (4)
C2A	0.0199 (5)	0.0166 (5)	0.0141 (5)	−0.0024 (4)	−0.0013 (4)	−0.0061 (4)
C3A	0.0238 (5)	0.0128 (5)	0.0181 (5)	−0.0014 (4)	−0.0022 (4)	−0.0042 (4)
C4A	0.0230 (5)	0.0155 (5)	0.0145 (5)	−0.0020 (4)	−0.0027 (4)	−0.0016 (4)
C5A	0.0155 (4)	0.0170 (5)	0.0153 (5)	−0.0027 (4)	−0.0002 (4)	−0.0052 (4)
O1A	0.0317 (4)	0.0138 (4)	0.0201 (4)	−0.0019 (3)	−0.0044 (3)	−0.0039 (3)
O2A	0.0339 (5)	0.0161 (4)	0.0150 (4)	−0.0027 (3)	−0.0025 (3)	−0.0050 (3)
O3A	0.0321 (4)	0.0161 (4)	0.0168 (4)	−0.0027 (3)	−0.0044 (3)	−0.0081 (3)
O4A	0.0282 (4)	0.0137 (4)	0.0181 (4)	−0.0040 (3)	0.0005 (3)	−0.0072 (3)
C6A	0.0151 (4)	0.0134 (5)	0.0149 (5)	−0.0024 (4)	0.0002 (4)	−0.0048 (4)
C7A	0.0163 (4)	0.0130 (5)	0.0151 (5)	−0.0024 (4)	0.0000 (4)	−0.0068 (4)
C8A	0.0223 (5)	0.0143 (5)	0.0150 (5)	−0.0025 (4)	−0.0003 (4)	−0.0046 (4)
C9A	0.0232 (5)	0.0193 (5)	0.0148 (5)	−0.0025 (4)	−0.0019 (4)	−0.0071 (4)
C10A	0.0208 (5)	0.0182 (5)	0.0189 (5)	−0.0031 (4)	−0.0008 (4)	−0.0107 (4)
C11A	0.0185 (5)	0.0137 (5)	0.0185 (5)	−0.0033 (4)	0.0003 (4)	−0.0065 (4)
C12A	0.0173 (5)	0.0163 (5)	0.0166 (5)	−0.0030 (4)	−0.0010 (4)	−0.0049 (4)
C13A	0.0174 (5)	0.0152 (5)	0.0170 (5)	−0.0017 (4)	−0.0003 (4)	−0.0082 (4)
Cl1B	0.03245 (15)	0.01553 (12)	0.01737 (13)	−0.00288 (10)	−0.00214 (10)	−0.00333 (10)
N1B	0.0214 (4)	0.0147 (4)	0.0153 (4)	−0.0026 (3)	0.0000 (3)	−0.0077 (4)
N2B	0.0317 (5)	0.0145 (4)	0.0157 (5)	−0.0021 (4)	−0.0024 (4)	−0.0062 (4)
C1B	0.0191 (5)	0.0180 (5)	0.0148 (5)	−0.0024 (4)	−0.0006 (4)	−0.0075 (4)
C2B	0.0203 (5)	0.0145 (5)	0.0150 (5)	−0.0022 (4)	−0.0002 (4)	−0.0048 (4)
C3B	0.0232 (5)	0.0161 (5)	0.0180 (5)	−0.0039 (4)	0.0011 (4)	−0.0098 (4)
C4B	0.0244 (5)	0.0171 (5)	0.0154 (5)	−0.0039 (4)	0.0003 (4)	−0.0087 (4)
C5B	0.0187 (5)	0.0164 (5)	0.0158 (5)	−0.0026 (4)	0.0009 (4)	−0.0080 (4)
O1B	0.0377 (5)	0.0162 (4)	0.0208 (4)	−0.0039 (4)	−0.0039 (4)	−0.0015 (3)
O2B	0.0468 (6)	0.0167 (4)	0.0135 (4)	0.0012 (4)	−0.0051 (4)	−0.0046 (3)
O3B	0.0350 (5)	0.0188 (4)	0.0133 (4)	−0.0041 (3)	−0.0026 (3)	−0.0063 (3)
O4B	0.0557 (6)	0.0146 (4)	0.0204 (4)	−0.0036 (4)	−0.0118 (4)	−0.0062 (3)
C6B	0.0170 (5)	0.0144 (5)	0.0149 (5)	−0.0021 (4)	0.0003 (4)	−0.0039 (4)
C7B	0.0200 (5)	0.0150 (5)	0.0143 (5)	−0.0033 (4)	−0.0008 (4)	−0.0060 (4)
C8B	0.0311 (6)	0.0152 (5)	0.0158 (5)	−0.0027 (4)	−0.0046 (4)	−0.0047 (4)
C9B	0.0322 (6)	0.0208 (6)	0.0166 (5)	−0.0015 (5)	−0.0072 (4)	−0.0073 (4)
C10B	0.0262 (6)	0.0197 (5)	0.0224 (6)	−0.0018 (4)	−0.0055 (4)	−0.0107 (5)
C11B	0.0218 (5)	0.0143 (5)	0.0207 (5)	−0.0023 (4)	−0.0018 (4)	−0.0062 (4)
C12B	0.0231 (5)	0.0172 (5)	0.0153 (5)	−0.0013 (4)	0.0007 (4)	−0.0038 (4)
C13B	0.0255 (5)	0.0163 (5)	0.0140 (5)	−0.0043 (4)	−0.0005 (4)	−0.0055 (4)
Cl1C	0.03599 (16)	0.01527 (13)	0.02110 (14)	−0.00401 (11)	−0.00132 (11)	−0.00209 (10)
N1C	0.0234 (5)	0.0146 (4)	0.0141 (4)	−0.0031 (3)	−0.0017 (3)	−0.0058 (3)
N2C	0.0339 (5)	0.0163 (5)	0.0139 (4)	−0.0053 (4)	−0.0028 (4)	−0.0050 (4)
C1C	0.0228 (5)	0.0163 (5)	0.0143 (5)	−0.0024 (4)	−0.0011 (4)	−0.0048 (4)
C2C	0.0218 (5)	0.0151 (5)	0.0154 (5)	−0.0028 (4)	0.0013 (4)	−0.0036 (4)
C3C	0.0258 (5)	0.0184 (5)	0.0197 (5)	−0.0053 (4)	0.0019 (4)	−0.0100 (4)
C4C	0.0272 (6)	0.0193 (5)	0.0159 (5)	−0.0055 (4)	0.0000 (4)	−0.0092 (4)
C5C	0.0196 (5)	0.0169 (5)	0.0145 (5)	−0.0034 (4)	0.0006 (4)	−0.0064 (4)
O1C	0.0328 (4)	0.0140 (4)	0.0182 (4)	−0.0034 (3)	−0.0033 (3)	−0.0067 (3)
O2C	0.0326 (4)	0.0150 (4)	0.0147 (4)	−0.0024 (3)	−0.0025 (3)	−0.0053 (3)
O3C	0.0397 (5)	0.0199 (4)	0.0137 (4)	−0.0033 (4)	−0.0043 (3)	−0.0060 (3)
O4C	0.0261 (4)	0.0210 (4)	0.0219 (4)	−0.0013 (3)	−0.0069 (3)	−0.0126 (3)
C6	0.0153 (4)	0.0131 (4)	0.0139 (5)	−0.0015 (3)	−0.0019 (3)	−0.0050 (4)
C7C	0.0156 (4)	0.0161 (5)	0.0140 (5)	−0.0024 (4)	−0.0020 (4)	−0.0066 (4)
C8C	0.0203 (5)	0.0141 (5)	0.0203 (5)	−0.0025 (4)	−0.0035 (4)	−0.0069 (4)
C9C	0.0226 (5)	0.0150 (5)	0.0192 (5)	−0.0028 (4)	−0.0039 (4)	−0.0026 (4)
C10C	0.0250 (5)	0.0201 (5)	0.0143 (5)	−0.0010 (4)	−0.0039 (4)	−0.0044 (4)
C11C	0.0215 (5)	0.0170 (5)	0.0144 (5)	−0.0014 (4)	−0.0031 (4)	−0.0067 (4)
C12C	0.0168 (5)	0.0146 (5)	0.0157 (5)	−0.0019 (4)	−0.0006 (4)	−0.0052 (4)
C13C	0.0170 (5)	0.0187 (5)	0.0152 (5)	−0.0011 (4)	−0.0025 (4)	−0.0083 (4)
O1X	0.0276 (4)	0.0166 (4)	0.0190 (4)	−0.0009 (3)	−0.0017 (3)	−0.0087 (3)
O2X	0.0203 (4)	0.0190 (4)	0.0179 (4)	−0.0035 (3)	−0.0032 (3)	−0.0099 (3)
O3X	0.0367 (5)	0.0150 (4)	0.0151 (4)	−0.0046 (3)	−0.0026 (3)	−0.0057 (3)
O4X	0.0341 (5)	0.0121 (4)	0.0180 (4)	−0.0022 (3)	−0.0065 (3)	−0.0048 (3)
C6X	0.0171 (5)	0.0157 (5)	0.0138 (5)	−0.0020 (4)	−0.0016 (4)	−0.0061 (4)
C7X	0.0173 (5)	0.0144 (5)	0.0143 (5)	−0.0015 (4)	−0.0015 (4)	−0.0061 (4)
C8X	0.0204 (5)	0.0180 (5)	0.0179 (5)	−0.0030 (4)	−0.0023 (4)	−0.0086 (4)
C9X	0.0232 (5)	0.0266 (6)	0.0175 (5)	−0.0042 (4)	−0.0047 (4)	−0.0100 (5)
C10X	0.0257 (6)	0.0238 (6)	0.0151 (5)	−0.0031 (4)	−0.0051 (4)	−0.0038 (4)
C11X	0.0236 (5)	0.0164 (5)	0.0160 (5)	−0.0040 (4)	−0.0027 (4)	−0.0033 (4)
C12X	0.0217 (5)	0.0119 (4)	0.0121 (4)	−0.0046 (4)	−0.0013 (4)	−0.0025 (4)
C13X	0.0173 (5)	0.0129 (5)	0.0158 (5)	−0.0031 (4)	−0.0007 (4)	−0.0054 (4)

### Geometric parameters (Å, °)

**Table:**

Cl1A—C2A	1.7311 (11)	C8B—H8BA	0.9300
N1A—C5A	1.3464 (14)	C9B—C10B	1.3865 (17)
N1A—C1A	1.3579 (14)	C9B—H9BA	0.9300
N1A—H1NA	0.899 (18)	C10B—C11B	1.3884 (17)
N2A—C5A	1.3320 (15)	C10B—H10D	0.9300
N2A—H2NA	0.896 (18)	C11B—H11D	0.9300
N2A—H3NA	0.888 (18)	Cl1C—C2C	1.7246 (12)
C1A—C2A	1.3575 (16)	N1C—C5C	1.3493 (14)
C1A—H1AA	0.9300	N1C—C1C	1.3532 (14)
C2A—C3A	1.4089 (15)	N1C—H1NC	0.94 (2)
C3A—C4A	1.3659 (16)	N2C—C5C	1.3276 (15)
C3A—H3AA	0.9300	N2C—H2NC	0.88 (2)
C4A—C5A	1.4199 (16)	N2C—H3NC	0.83 (2)
C4A—H4AA	0.9300	C1C—C2C	1.3603 (16)
O1A—C12A	1.2315 (14)	C1C—H1CA	0.9300
O2A—C12A	1.2927 (14)	C2C—C3C	1.4125 (16)
O2A—H1OA	0.8626	C3C—C4C	1.3649 (17)
O3A—C13A	1.2748 (14)	C3C—H3CA	0.9300
O4A—C13A	1.2453 (14)	C4C—C5C	1.4214 (16)
C6A—C11A	1.4007 (15)	C4C—H4CA	0.9300
C6A—C7A	1.4201 (14)	O1C—C12C	1.2388 (14)
C6A—C12A	1.5212 (15)	O2C—C12C	1.2873 (13)
C7A—C8A	1.3981 (15)	O2C—H2C	0.8200
C7A—C13A	1.5237 (15)	O3C—C13C	1.2814 (14)
C8A—C9A	1.3849 (16)	O4C—C13C	1.2400 (14)
C8A—H8AA	0.9300	C6—C11C	1.3986 (15)
C9A—C10A	1.3902 (16)	C6—C7C	1.4218 (15)
C9A—H9AA	0.9300	C6—C12C	1.5191 (15)
C10A—C11A	1.3882 (16)	C7C—C8C	1.4009 (15)
C10A—H10C	0.9300	C7C—C13C	1.5186 (15)
C11A—H11C	0.9300	C8C—C9C	1.3852 (16)
Cl1B—C2B	1.7264 (11)	C8C—H8CA	0.9300
N1B—C5B	1.3468 (14)	C9C—C10C	1.3839 (17)
N1B—C1B	1.3543 (15)	C9C—H9CA	0.9300
N1B—H1NB	0.92 (2)	C10C—C11C	1.3854 (16)
N2B—C5B	1.3349 (15)	C10C—H10B	0.9300
N2B—H2NB	0.865 (19)	C11C—H11B	0.9300
N2B—H3NB	0.865 (19)	O1X—C12X	1.2170 (14)
C1B—C2B	1.3618 (15)	O2X—C12X	1.3234 (14)
C1B—H1BA	0.9300	O2X—H1OX	0.88 (2)
C2B—C3B	1.4125 (15)	O3X—C13X	1.2088 (14)
C3B—C4B	1.3666 (16)	O4X—C13X	1.3277 (13)
C3B—H3BA	0.9300	O4X—H2OX	0.83 (2)
C4B—C5B	1.4169 (15)	C6X—C11X	1.3938 (15)
C4B—H4BA	0.9300	C6X—C7X	1.4043 (15)
O1B—C12B	1.2266 (15)	C6X—C12X	1.5023 (15)
O2B—C12B	1.2990 (15)	C7X—C8X	1.3971 (15)
O2B—H1OB	0.8922	C7X—C13X	1.4928 (15)
O3B—C13B	1.2698 (14)	C8X—C9X	1.3900 (16)
O4B—C13B	1.2480 (14)	C8X—H8XA	0.9300
C6B—C11B	1.4004 (16)	C9X—C10X	1.3903 (18)
C6B—C7B	1.4198 (15)	C9X—H9XA	0.9300
C6B—C12B	1.5208 (15)	C10X—C11X	1.3940 (16)
C7B—C8B	1.3999 (15)	C10X—H10A	0.9300
C7B—C13B	1.5242 (16)	C11X—H11A	0.9300
C8B—C9B	1.3829 (16)		
			
C5A—N1A—C1A	122.94 (10)	C11B—C10B—H10D	120.4
C5A—N1A—H1NA	120.8 (11)	C10B—C11B—C6B	122.47 (11)
C1A—N1A—H1NA	116.2 (11)	C10B—C11B—H11D	118.8
C5A—N2A—H2NA	118.7 (12)	C6B—C11B—H11D	118.8
C5A—N2A—H3NA	121.1 (12)	O1B—C12B—O2B	120.49 (11)
H2NA—N2A—H3NA	120.1 (16)	O1B—C12B—C6B	119.76 (11)
C2A—C1A—N1A	120.19 (10)	O2B—C12B—C6B	119.73 (10)
C2A—C1A—H1AA	119.9	O4B—C13B—O3B	122.28 (11)
N1A—C1A—H1AA	119.9	O4B—C13B—C7B	117.01 (10)
C1A—C2A—C3A	119.33 (10)	O3B—C13B—C7B	120.71 (10)
C1A—C2A—Cl1A	119.37 (9)	C5C—N1C—C1C	122.59 (10)
C3A—C2A—Cl1A	121.30 (9)	C5C—N1C—H1NC	119.0 (13)
C4A—C3A—C2A	119.73 (10)	C1C—N1C—H1NC	118.4 (13)
C4A—C3A—H3AA	120.1	C5C—N2C—H2NC	116.2 (12)
C2A—C3A—H3AA	120.1	C5C—N2C—H3NC	119.9 (13)
C3A—C4A—C5A	119.92 (10)	H2NC—N2C—H3NC	123.7 (17)
C3A—C4A—H4AA	120.0	N1C—C1C—C2C	120.46 (10)
C5A—C4A—H4AA	120.0	N1C—C1C—H1CA	119.8
N2A—C5A—N1A	118.85 (10)	C2C—C1C—H1CA	119.8
N2A—C5A—C4A	123.27 (10)	C1C—C2C—C3C	119.41 (10)
N1A—C5A—C4A	117.88 (10)	C1C—C2C—Cl1C	119.03 (9)
C12A—O2A—H1OA	110.8	C3C—C2C—Cl1C	121.55 (9)
C11A—C6A—C7A	118.17 (10)	C4C—C3C—C2C	119.35 (11)
C11A—C6A—C12A	113.49 (9)	C4C—C3C—H3CA	120.3
C7A—C6A—C12A	128.33 (10)	C2C—C3C—H3CA	120.3
C8A—C7A—C6A	118.26 (10)	C3C—C4C—C5C	120.09 (11)
C8A—C7A—C13A	113.54 (9)	C3C—C4C—H4CA	120.0
C6A—C7A—C13A	128.20 (10)	C5C—C4C—H4CA	120.0
C9A—C8A—C7A	122.76 (10)	N2C—C5C—N1C	118.63 (10)
C9A—C8A—H8AA	118.6	N2C—C5C—C4C	123.31 (11)
C7A—C8A—H8AA	118.6	N1C—C5C—C4C	118.06 (10)
C8A—C9A—C10A	119.01 (11)	C12C—O2C—H2C	109.5
C8A—C9A—H9AA	120.5	C11C—C6—C7C	118.23 (10)
C10A—C9A—H9AA	120.5	C11C—C6—C12C	113.09 (9)
C11A—C10A—C9A	119.41 (10)	C7C—C6—C12C	128.68 (10)
C11A—C10A—H10C	120.3	C8C—C7C—C6	118.07 (10)
C9A—C10A—H10C	120.3	C8C—C7C—C13C	113.93 (9)
C10A—C11A—C6A	122.38 (10)	C6—C7C—C13C	127.93 (10)
C10A—C11A—H11C	118.8	C9C—C8C—C7C	122.41 (11)
C6A—C11A—H11C	118.8	C9C—C8C—H8CA	118.8
O1A—C12A—O2A	120.63 (11)	C7C—C8C—H8CA	118.8
O1A—C12A—C6A	118.80 (10)	C10C—C9C—C8C	119.41 (11)
O2A—C12A—C6A	120.53 (10)	C10C—C9C—H9CA	120.3
O4A—C13A—O3A	122.55 (10)	C8C—C9C—H9CA	120.3
O4A—C13A—C7A	116.75 (10)	C9C—C10C—C11C	119.34 (11)
O3A—C13A—C7A	120.70 (10)	C9C—C10C—H10B	120.3
C5B—N1B—C1B	122.49 (10)	C11C—C10C—H10B	120.3
C5B—N1B—H1NB	120.8 (13)	C10C—C11C—C6	122.45 (11)
C1B—N1B—H1NB	116.6 (13)	C10C—C11C—H11B	118.8
C5B—N2B—H2NB	122.8 (12)	C6—C11C—H11B	118.8
C5B—N2B—H3NB	114.2 (12)	O1C—C12C—O2C	120.72 (10)
H2NB—N2B—H3NB	123.0 (17)	O1C—C12C—C6	117.77 (10)
N1B—C1B—C2B	120.52 (10)	O2C—C12C—C6	121.51 (10)
N1B—C1B—H1BA	119.7	O4C—C13C—O3C	121.99 (10)
C2B—C1B—H1BA	119.7	O4C—C13C—C7C	117.77 (10)
C1B—C2B—C3B	119.40 (10)	O3C—C13C—C7C	120.24 (10)
C1B—C2B—Cl1B	118.85 (9)	C12X—O2X—H1OX	109.1 (12)
C3B—C2B—Cl1B	121.73 (9)	C13X—O4X—H2OX	108.6 (14)
C4B—C3B—C2B	119.05 (10)	C11X—C6X—C7X	119.87 (10)
C4B—C3B—H3BA	120.5	C11X—C6X—C12X	117.43 (10)
C2B—C3B—H3BA	120.5	C7X—C6X—C12X	122.66 (9)
C3B—C4B—C5B	120.41 (10)	C8X—C7X—C6X	119.68 (10)
C3B—C4B—H4BA	119.8	C8X—C7X—C13X	121.01 (10)
C5B—C4B—H4BA	119.8	C6X—C7X—C13X	119.15 (9)
N2B—C5B—N1B	119.18 (10)	C9X—C8X—C7X	120.18 (11)
N2B—C5B—C4B	122.69 (10)	C9X—C8X—H8XA	119.9
N1B—C5B—C4B	118.13 (10)	C7X—C8X—H8XA	119.9
C12B—O2B—H1OB	110.9	C8X—C9X—C10X	119.94 (11)
C11B—C6B—C7B	118.19 (10)	C8X—C9X—H9XA	120.0
C11B—C6B—C12B	113.14 (10)	C10X—C9X—H9XA	120.0
C7B—C6B—C12B	128.66 (10)	C9X—C10X—C11X	120.44 (11)
C8B—C7B—C6B	118.08 (10)	C9X—C10X—H10A	119.8
C8B—C7B—C13B	113.18 (10)	C11X—C10X—H10A	119.8
C6B—C7B—C13B	128.74 (10)	C6X—C11X—C10X	119.82 (11)
C9B—C8B—C7B	122.76 (11)	C6X—C11X—H11A	120.1
C9B—C8B—H8BA	118.6	C10X—C11X—H11A	120.1
C7B—C8B—H8BA	118.6	O1X—C12X—O2X	124.43 (10)
C8B—C9B—C10B	119.23 (11)	O1X—C12X—C6X	123.11 (10)
C8B—C9B—H9BA	120.4	O2X—C12X—C6X	112.32 (9)
C10B—C9B—H9BA	120.4	O3X—C13X—O4X	123.32 (10)
C9B—C10B—C11B	119.25 (11)	O3X—C13X—C7X	122.29 (10)
C9B—C10B—H10D	120.4	O4X—C13X—C7X	114.38 (9)
			
C5A—N1A—C1A—C2A	−0.11 (17)	C7B—C6B—C12B—O2B	3.60 (18)
N1A—C1A—C2A—C3A	0.01 (17)	C8B—C7B—C13B—O4B	−7.92 (16)
N1A—C1A—C2A—Cl1A	179.51 (8)	C6B—C7B—C13B—O4B	171.08 (12)
C1A—C2A—C3A—C4A	−0.43 (17)	C8B—C7B—C13B—O3B	171.62 (11)
Cl1A—C2A—C3A—C4A	−179.91 (9)	C6B—C7B—C13B—O3B	−9.37 (19)
C2A—C3A—C4A—C5A	0.91 (17)	C5C—N1C—C1C—C2C	−1.30 (17)
C1A—N1A—C5A—N2A	−179.42 (10)	N1C—C1C—C2C—C3C	−0.34 (17)
C1A—N1A—C5A—C4A	0.59 (16)	N1C—C1C—C2C—Cl1C	−179.82 (9)
C3A—C4A—C5A—N2A	179.02 (11)	C1C—C2C—C3C—C4C	0.94 (18)
C3A—C4A—C5A—N1A	−0.99 (16)	Cl1C—C2C—C3C—C4C	−179.60 (9)
C11A—C6A—C7A—C8A	−0.66 (15)	C2C—C3C—C4C—C5C	0.03 (18)
C12A—C6A—C7A—C8A	178.68 (10)	C1C—N1C—C5C—N2C	−177.75 (11)
C11A—C6A—C7A—C13A	178.97 (10)	C1C—N1C—C5C—C4C	2.23 (17)
C12A—C6A—C7A—C13A	−1.68 (18)	C3C—C4C—C5C—N2C	178.42 (12)
C6A—C7A—C8A—C9A	−0.18 (17)	C3C—C4C—C5C—N1C	−1.57 (17)
C13A—C7A—C8A—C9A	−179.86 (10)	C11C—C6—C7C—C8C	−2.71 (15)
C7A—C8A—C9A—C10A	0.61 (18)	C12C—C6—C7C—C8C	177.49 (10)
C8A—C9A—C10A—C11A	−0.19 (17)	C11C—C6—C7C—C13C	173.98 (10)
C9A—C10A—C11A—C6A	−0.68 (17)	C12C—C6—C7C—C13C	−5.82 (18)
C7A—C6A—C11A—C10A	1.11 (16)	C6—C7C—C8C—C9C	2.13 (16)
C12A—C6A—C11A—C10A	−178.34 (10)	C13C—C7C—C8C—C9C	−175.01 (10)
C11A—C6A—C12A—O1A	−10.47 (15)	C7C—C8C—C9C—C10C	0.30 (18)
C7A—C6A—C12A—O1A	170.16 (11)	C8C—C9C—C10C—C11C	−2.10 (18)
C11A—C6A—C12A—O2A	167.19 (10)	C9C—C10C—C11C—C6	1.46 (18)
C7A—C6A—C12A—O2A	−12.18 (17)	C7C—C6—C11C—C10C	0.99 (17)
C8A—C7A—C13A—O4A	4.03 (14)	C12C—C6—C11C—C10C	−179.18 (10)
C6A—C7A—C13A—O4A	−175.62 (10)	C11C—C6—C12C—O1C	2.83 (14)
C8A—C7A—C13A—O3A	−175.22 (10)	C7C—C6—C12C—O1C	−177.36 (11)
C6A—C7A—C13A—O3A	5.13 (17)	C11C—C6—C12C—O2C	−177.53 (10)
C5B—N1B—C1B—C2B	−0.22 (17)	C7C—C6—C12C—O2C	2.28 (17)
N1B—C1B—C2B—C3B	0.16 (17)	C8C—C7C—C13C—O4C	4.56 (15)
N1B—C1B—C2B—Cl1B	178.83 (8)	C6—C7C—C13C—O4C	−172.25 (11)
C1B—C2B—C3B—C4B	−0.02 (17)	C8C—C7C—C13C—O3C	−175.82 (10)
Cl1B—C2B—C3B—C4B	−178.65 (9)	C6—C7C—C13C—O3C	7.38 (17)
C2B—C3B—C4B—C5B	−0.07 (17)	C11X—C6X—C7X—C8X	1.67 (16)
C1B—N1B—C5B—N2B	−179.22 (11)	C12X—C6X—C7X—C8X	−175.84 (10)
C1B—N1B—C5B—C4B	0.13 (16)	C11X—C6X—C7X—C13X	−173.77 (10)
C3B—C4B—C5B—N2B	179.35 (11)	C12X—C6X—C7X—C13X	8.72 (16)
C3B—C4B—C5B—N1B	0.02 (17)	C6X—C7X—C8X—C9X	0.65 (17)
C11B—C6B—C7B—C8B	1.11 (16)	C13X—C7X—C8X—C9X	176.00 (10)
C12B—C6B—C7B—C8B	−178.24 (11)	C7X—C8X—C9X—C10X	−2.29 (18)
C11B—C6B—C7B—C13B	−177.86 (11)	C8X—C9X—C10X—C11X	1.62 (19)
C12B—C6B—C7B—C13B	2.79 (19)	C7X—C6X—C11X—C10X	−2.34 (17)
C6B—C7B—C8B—C9B	0.24 (18)	C12X—C6X—C11X—C10X	175.30 (11)
C13B—C7B—C8B—C9B	179.36 (12)	C9X—C10X—C11X—C6X	0.70 (18)
C7B—C8B—C9B—C10B	−1.2 (2)	C11X—C6X—C12X—O1X	64.87 (15)
C8B—C9B—C10B—C11B	0.80 (19)	C7X—C6X—C12X—O1X	−117.57 (13)
C9B—C10B—C11B—C6B	0.58 (19)	C11X—C6X—C12X—O2X	−110.99 (11)
C7B—C6B—C11B—C10B	−1.54 (17)	C7X—C6X—C12X—O2X	66.58 (14)
C12B—C6B—C11B—C10B	177.91 (11)	C8X—C7X—C13X—O3X	−160.33 (11)
C11B—C6B—C12B—O1B	2.92 (16)	C6X—C7X—C13X—O3X	15.04 (16)
C7B—C6B—C12B—O1B	−177.70 (12)	C8X—C7X—C13X—O4X	18.32 (15)
C11B—C6B—C12B—O2B	−175.78 (11)	C6X—C7X—C13X—O4X	−166.30 (10)

### Hydrogen-bond geometry (Å, °)

**Table:**

*D*—H···*A*	*D*—H	H···*A*	*D*···*A*	*D*—H···*A*
O2C—H2C···O3C	0.82	1.58	2.4004 (13)	177
O2A—H1OA···O3A	0.86	1.55	2.4123 (13)	177
O2B—H1OB···O3B	0.89	1.52	2.4126 (14)	178
O2X—H1OX···O4C	0.88 (2)	1.76 (2)	2.6091 (12)	163 (2)
O4X—H2OX···O1A^i^	0.83 (2)	1.86 (2)	2.6880 (13)	177 (2)
N2A—H2NA···O1B^ii^	0.895 (18)	2.018 (18)	2.9095 (14)	173.4 (19)
N1B—H1NB···O4A^iii^	0.92 (2)	1.69 (2)	2.5938 (14)	169 (2)
N2B—H2NB···O3A^iii^	0.87 (2)	2.12 (2)	2.9570 (14)	161.8 (17)
N1C—H1NC···O4B	0.94 (2)	1.69 (2)	2.6169 (16)	168 (2)
N2C—H3NC···O3B	0.83 (2)	2.11 (2)	2.9279 (15)	168.4 (19)
C4A—H4AA···O2B^ii^	0.93	2.26	3.1614 (14)	164

Symmetry codes: (i) *x*, *y*−1, *z*; (ii) −*x*+1, −*y*+1, −*z*; (iii) −*x*+1, −*y*+1, −*z*+1.
